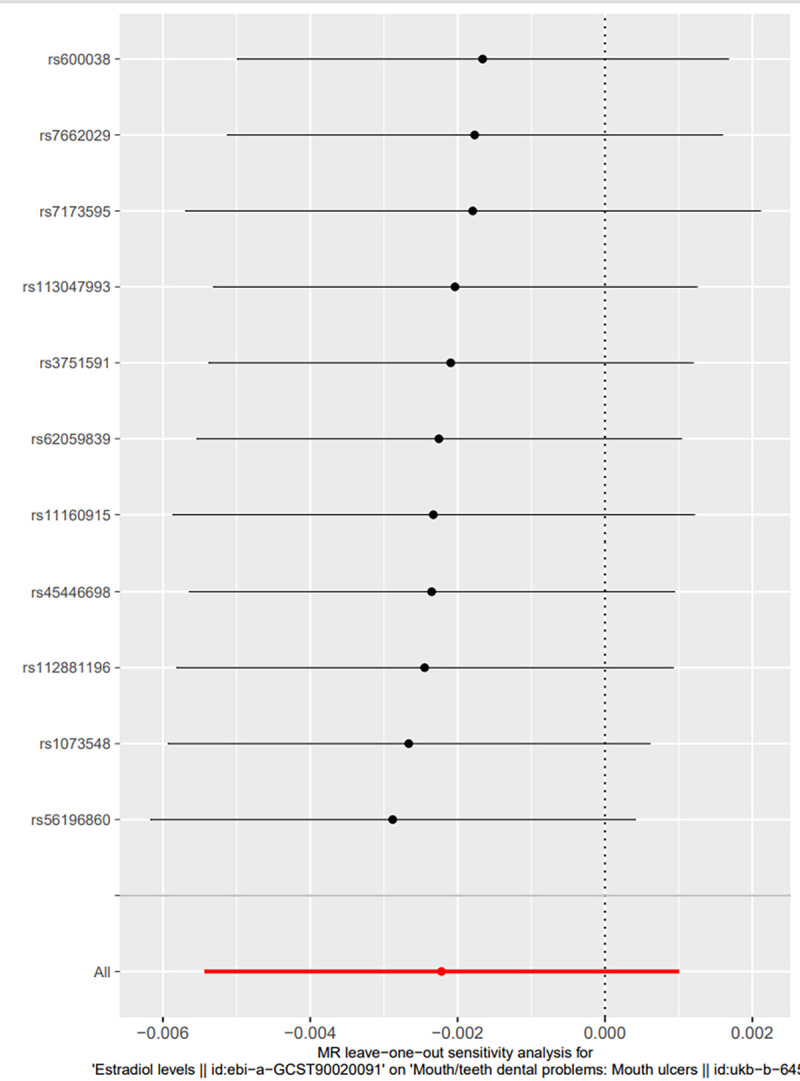# Causal associations between estradiol and mouth ulcers: A Mendelian randomization study

**DOI:** 10.1097/MD.0000000000037989

**Published:** 2024-04-26

**Authors:** Yaqian Zhang, Kunting Zhong, Weiyu Liang, Ruihanqiu Liu, Weiling Qu, Yan Lu

**Affiliations:** a Research Centre of Basic Integrative Medicine, School of Basic Medical Sciences, Guangzhou University of Chinese Medicine, Guangzhou, Guangdong Province, China.

**Keywords:** causal relationship, estradiol, mendelian randomization, mouth ulcers

## Abstract

People have difficulty in eating and speaking when they are suffering from mouth ulcers. Some studies suggest that estradiol is associated with the development and treatment of mouth ulcers, while some do not. To clarify the effect of estradiol on mouth ulcers, we performed 2-sample Mendelian randomization and multivariable Mendelian randomization (MVMR) analysis to evaluate their relationship. Data were obtained from the IEU OpenGWAS project and UK biobank, including male estradiol dataset (case/controls = 13,367/134,323), female estradiol dataset (case/controls = 37,461/126,524), mouth ulcers dataset (case/controls = 47,102/414,011). The causal associations were estimated by MR-Egger, weighted median, inverse-variance weighted (IVW) method, simple mode, and weighted mode. Cochran Q test, MR-Egger intercept test, MR-PRESSO tests, and leave-one-out analysis were used to examine sensitivity analyses. The MVMR controlling for depression, anxiety or panic attacks, severe stress and adjustment disorders was used to assess the effect of estradiol on mouth ulcers. Through screening, 13 single nucleotide polymorphisms (SNPs) of males and 2 SNPs of females in estradiol were used for harmonizing and MR analysis. The 2-sample MR analysis showed no causal association between estradiol of males and mouth ulcers (IVW, OR: 0.998, 95% confidence interval [95% CI]: 0.995–1.001, *P* = .18). Similar results were obtained between estradiol of females and mouth ulcers (IVW, OR: 1.000, 95% CI: 0.988–1.012, *P* = .97). No pleiotropy and heterogeneity were found and the results were robust (*P* > .05). After adjusting for the potential effects of confounders, estradiol of males and mouth ulcers still showed no causal association through MVMR analysis (*P* = .081). While MVMR analysis showed that the causal relationship between estradiol and mouth ulcers in women could not be statistical for the small number of SNPs. There was no evidence of a causal relationship between estradiol and mouth ulcers. The strategy of treating mouth ulcers with estradiol still needs to be confirmed by more studies.

## 1. Introduction

Mouth ulcers manifest a chronic inflammatory injury in the oral mucosa. With an incidence varying between 5% and 66% in the general population, mouth ulcers, especially recurrent aphthous stomatitis are one of the most common oral diseases.^[1]^ The ulcer lesions can be uncomfortable at rest, and pain is most intense during foreign body contact, which impacts people eating and speaking.^[2]^ There are complex and varied factors that may be involved in the etiology of mouth ulcers, like physical injury, stress, hormonal imbalance, nutritional deficiency, genetic factors, and infection.^[3–5]^ Regarding the etiology and extent of the disease, patients with individual differences choose different treatments, resulting in a situation where there is no uniform, proven treatment way for mouth ulcers at present.^[6]^

Estrogens, derived from cholesterol, are steroid hormones. Estradiol is one of the principal natural estrogens and is a main hormone in women.^[7]^ Estrogen receptors have been found in the oral mucosa, for instance, the mRNA of estrogen receptors has been detected by Reverse Transcription-Polymerase Chain Reaction in buccal mucosa samples.^[8,9]^ Some studies confirmed that estradiol levels were strongly linked to the development of mouth ulcers by revealing that oral ulcers often recurred in women before or during menstruation when a phase of low estrogen levels and estradiol had a therapeutic role in oral ulcers.^[10–12]^ The development of mouth ulcers has an association with the loss or apoptosis of oral epithelium, and the damage of epithelium can explain the quality of life of those who suffer from mouth ulcers.^[13,14]^ Estrogens could increase epithelial keratinization and stimulate proliferation, which may involve the onset and healing of mouth ulcers. Correspondingly, postmenopausal women, a common population with mouth ulcers, have a reduction in keratinization of marginal gingival epithelium with plasma estrogen levels declining.^[15]^ Additionally, estrogens can mediate the actions of gingival and periodontal tissue fibroblasts which with high intracellular fibroblast growth factors, like fibroblast growth factor 2, could mediate the cytoprotective effect of epithelial cells, and be helpful for oral wound healing.^[16,17]^ These suggested that estradiol may act as a trigger for mouth ulcers. However, the evidence supporting this association was inconsistent with other studies which had shown no significant association.^[18]^ There are conflicting accounts of the role of estradiol in mouth ulcers maybe due to the lack of clinical large sample size trials. In addition, some confounders containing unmeasured risk factors in existing trials cannot be completely ruled out. Moreover, the mechanism for estrogen treatment of mouth ulcers has not been identified to date. Thus, further studies are needed to elucidate whether there is a causal or other relationship between mouth ulcers and estradiol.

Mendelian randomization (MR) is a genetic epidemiology design which is an effective method to understand whether risk factors have a causation with the development of 1 disease.^[19]^ Using single nucleotide polymorphisms (SNPs), derived from studies of large sample sizes, as instrumental variables, the 2-sample MR analysis can mitigate unobserved confounding that clinical trials and experiments cannot completely control. Multivariable MR could control for other exposure factors to estimate the effect of 1 exposure factor. Because the gene encoding estrogen receptor beta is linked to depression, and the drop in estrogen may increase the risk of emotions, like anxiety, stress, and depression, which are more common in patients with mouth ulcers than in healthy people,^[20,21]^ depression, anxiety or panic attacks, severe stress and adjustment disorders were considered as confounding factors.

In this study, we conducted 2-sample MR and multivariable MR to evaluate the causal effect of estradiol on mouth ulcers after controlling some confounders..

## 2. Materials and methods

### 2.1. Study design

**Figure 1.**
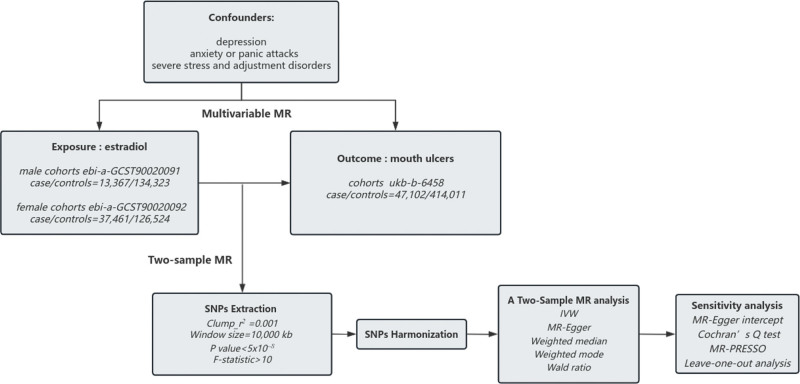
Workflow of Mendelian randomization in this study.

The analysis was based on the following 3 assumptions. The SNPs should be associated with the exposure (estradiol), and the *P* value of SNPs we obtained from the GWAS < 5 × 10^–8^; the SNPs should be independent of confounders. Clumping SNPs of estradiol with linkage disequilibrium (r^2^ = 0.001 at a 10,000 kb window), we confirmed the independence of the chosen genetic variants.; the SNPs should not directly affect the outcome (mouth ulcers). The workflow of this study is shown in Figure 1.

### 2.2. Data sources

The genetic instruments of estradiol and mouth ulcers were obtained from the IEU Open GWAS project (https://gwas.mrcieu.ac.uk/) and UK biobank. The population of them were both European. The data of estradiol had been measured by a 2-step competitive analysis on a Beckman Coulter Unicel Dxl 800 and its measurements included from the blood samples.^[22]^ There were 7488,193 SNPs, 163,985 sample sizes, 126,524 controls, and 37,461 cases for estradiol cohorts in females. About the male cohorts, the number of SNPs is 7489,424, with 147,690 sample sizes, 134,323 controls, and 13,367 cases. Information on mouth ulcers was collected retrospectively by a touchscreen questionnaire requesting participants to answer “Do you have any of the following? (You can select more than 1 answer).” The answers contained “Mouth ulcers, Painful gums, Bleeding gums, Loose teeth, Toothache, Dentures, None of the above, Prefer not to answer.” There were 9851,867 SNPs, 461,113 sample sizes, 414,011 controls, and 47,102 cases of mouth ulcers. The estradiol SNP data of female cohorts ebi-a-GCST90020092 and male cohorts ebi-a-GCST90020091 were analyzed with mouth ulcers SNPs data of cohorts ukb-b-6458. The characteristics of the SNPs of both estradiol and mouth ulcers are shown in Table 1. Additionally, the information on confounders’ SNPs is shown in the supplement Table 1, http://links.lww.com/MD/M310.

**Table 1:** The characteristics of the SNPs of both estradiol and mouth ulcers.

Traits	Population	Sex	Sample Size (case/controls)	Database	PubMed ID
Estradiol	European	Males	13,367/134,323	ebi-a-GCST90020091	34255042
		Females	37,461/126,524	ebi-a-GCST90020092	34255042
Mouth ulcers	European	Males and Females	47,102/414,011	ukb-b-6458	-*

SNP = single nucleotide polymorphism.

* The data source of Mouth ulcers came from https://gwas.mrcieu.ac.uk/datasets/ukb-b-6458.

### 2.3. Statistical analysis

In this study, after employing the harmonizing process to exclude the ambiguous and palindromic SNPs (A/T or G/C) from those extracting SNPs, we used MR analysis methods, including MR-Egger, weighted median, inverse-variance weighted (IVW), Simple mode, and weighted mode. We used the value of Cochrane Q and the funnel plot to evaluate the heterogeneity. We applied the intercept test from MR-Egger to assess horizontal pleiotropy. The MR-PRESSO test was used to detect possible outliers and generate corrected estimates after removing them. The NbDistribution we set was 3000. The robustness of MR analysis results was assessed by a leave-one-out analysis through any outlier SNP. F-statistics (F = R^2^ (N-2)/(1- R^2^), R^2^ = 2 × (1-MAF) × MAF×(β) ^2^) were calculated to assess the strength of each SNP. In multivariable Mendelian randomization (MVMR), estradiol, depression, anxiety or panic attacks, reaction to severe stress and adjustment disorders were used as exposures simultaneously to explore their effects on mouth ulcers. All MR analyses involved in this study depended on the “TwoSampleMR” R package which was performed by R (version 4.2.1), and *P* value < .05 was statistically significant.

## 3. Results

**Figure 2.**
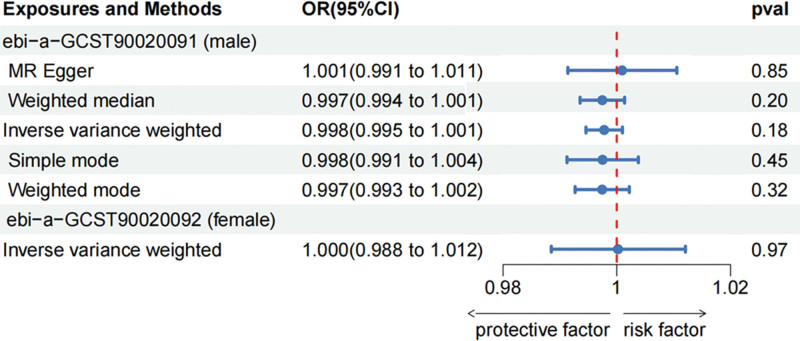
Result of the 2-sample MR analysis. MR = Mendelian randomization.

The 2-sample MR analysis showed that there was no causal association between estradiol and mouth ulcers (Fig. 2). F-statistics for each SNP were >10, so there was less possibility of weak instrumental bias in this study. After harmonizing, the estradiol of males has 11 SNPs as instruments which we used random effects models of IVW to analyze, while in the estradiol of females, for only 2 SNPs as instruments, we used fixed effects models to generate effect estimates. As for the male, the IVW analysis showed little association between estradiol levels and mouth ulcers (OR: 0.998, 95% confidence interval [95% CI]: 0.995–1.001, *P* = .18), which was consistent with 4 other different MR methods (all *P* values > .05). There was little evidence of heterogeneity or pleiotropy in the IVW analyses referring to Cochran Q statistic and MR-Egger intercepts. Besides, no outlier was found in the MR-PRESSO test, and the *P* value of the Global Test was 0.659. The leave-one-out analysis (S1 Fig, http://links.lww.com/MD/M309) proved that the results were quite robust and were not driven by any single SNP. For the results of the female, we only obtained 2 matching SNPs (rs45446698, rs16991615) from the database for the final analysis. Thus, the results of MR analysis just showed the Inverse-variance-weighted analysis (OR: 1.000, 95% CI: 0.988–1.012, *P* = .97). The pleiotropy and leave-one-out analysis cannot be calculated for the small numbers of SNPs. Similarly, we cannot detect outlier SNPs by the MR-PRESSO method. No obvious heterogeneity was shown either. The MVMR analysis in males, showed 13 nSNPs, demonstrating that estradiol was still not significantly associated with the risk of mouth ulcers after adjustment (OR: 0.998, 95% CI: 0.995–1.000, *P* = .081). About the females, the MVMR analysis could not be implemented because it only showed 3 nSNPs which are too small to statistic.

MR analysis did not show any evidence that increased estradiol levels of males and females are causally associated with a decreased risk of mouth ulcers. There was a noncausal association between estradiol and mouth ulcers.

## 4. Discussion

The present study suggested that estradiol, whether of man or woman, had no causal association with mouth ulcers after considering the effects of depression, anxiety or panic attacks, severe stress and adjustment disorders by integrating the results of 2-sample MR and multivariable MR analysis.

Some previous studies had established a close relationship between estradiol and mouth ulcers. P M Bishop found that estrogen therapy healed the recurrent aphthous ulceration effectively in 43 women, 33 of whom had a clearly defined link with the menstrual cycle.^[12]^ Besides, Kazuhide Seko thought that estrogen replacement may compensate for the reduction of mucosal thickness in ovariectomized rats.^[23]^ This evidence showed that estrogen has a strong link with mouth ulcers and it might have a therapeutic effect on mouth ulcers. In contrast, other studies demonstrated no significant association between them. A cross-sectional study did not find significantly abnormal estradiol levels in women with recurrent aphthous stomatitis.^[18]^ And B E McCartan thought there was no association between aphthous stomatitis and the premenstrual period, pregnancy, or menopause with fluctuating hormonal status.^[24]^ These studies are not consistent in supporting the association between estradiol and mouth ulcers. Therefore, to clarify their relationship, we conducted a 2-sample MR analysis to evaluate whether estradiol causally influenced mouth ulcer incidence.

In this study, no clear evidence supported that increased estradiol levels were causally associated with decreased mouth ulcer risk. Although our study showed no causality of estradiol on mouth ulcers, a sudden change in estradiol (e.g., before or during menstruation) might have disrupted the homeostasis state in the body, like the immunoinflammatory system, affecting the occurrence of mouth ulcers. At present, the pathogenesis of mouth ulcers remains unclear and some studies think that mouth ulcers are the process of immune-mediated mucosal destruction, inflammation, and proliferative healing.^[25,26]^ The pathogenesis of mouth ulcers contains T-cell immune dysregulation manifesting in that Th1-type hyperimmune response causes inflammation and ulcerations with cytokines like interleukin-6, tumor necrosis factor-alpha, and interferon-gamma.^[27]^ In recent years, it has been found that estrogens and their 3 estrogen receptors (ERα, ERβ, and G-protein coupled receptor) have roles in regulating the activity of different immune cells.^[28]^ In addition, the production of cytokines has been shown to be regulated by sex steroid hormones.^[29]^ Estrogen may act on mouth ulcers by influencing immune-inflammatory processes. Increased estradiol can play a role in antiinflammatory action via suppressing interferon and nuclear factor kappa B signaling.^[30,31]^ Moreover, high levels of estradiol prefer to shift T helper cells toward the repair skewed Th2 transcription profile relating to IL-4 during pregnancy.^[32]^ Therefore, we supposed that the shift of T helper cells toward the Th1 type as well as cytokines increasing during sudden deficiency of estradiol affected the development of mouth ulcers, and the T helper cells toward the Th2 type after estradiol being supplemented reversed the state. Thus, estradiol and mouth ulcers may have some correlation but not causation. Besides, whether estrogen affects mouth ulcers in this way is still worth exploring.

In addition, we cannot rule out another possibility. We suspected that it may not be estradiol but other sex hormones that did the job in mouth ulcers. It was quite possible that low estrogen levels before or during menstruation for the female physiological period created the illusion that estrogen was working. Meanwhile, some studies did not find any obvious changing level of estradiol in patients during mouth ulcers. Fatemeh Lavaee found that nonmenopause women with recurrent aphthous stomatitis had normal estradiol levels as well as abnormal serum Dehydroepiandrosterone sulfate, testosterone, and progesterone levels.^[18]^ Christopher G Engeland observed that no strong association between healing times and estradiol or progesterone levels in patients with wounds on the hard oral palate, but testosterone may impact healing.^[33]^ In addition, it is confirmed that female patients with cyclical mouth ulcers were cured with progestogen or testosterone, and nowadays the common medications used for treatment are corticosteroids, antibiotics, and analgesics.^[27,34,35]^ These ideas indicated that estradiol may not be a direct factor in the development of mouth ulcers. It may be the result of other hormones or a combination of sex hormones. Thus, the approach of estrogen treating mouth ulcers is worth skeptical and it still needs to be demonstrated by in-depth basic experiments and some clinical trials with large sample sizes.

Since only 2 SNPs were significantly associated with female estradiol, sensitivity studies were not well conducted. SNP rs45446698 and rs16991615 were closely related to the risk of breast cancer.^[36,37]^ Meanwhile, it had been reported an association between mouth ulcers and breast cancer since breast cancer patients had a difference in the oral microbiome with healthy controls, and the oral microbiome may relate to the development of mouth ulcers.^[38,39]^ However, Zeni Wu observed no association between self-reported mouth ulcers with the risk of breast cancer.^[40]^ No evidence showed that breast cancers were a factor in the development of mouth ulcers. Thus, breast cancer might not violate our findings.

We analyzed the relationship from a perspective of genetics focusing on lifetime effects rather than short-term effects, which avoided effectively confounding bias for randomly assigned SNPs at conception. However, our results also should be interpreted in conjunction with its limitations. First, we cannot explore the potential nonlinear association between estradiol levels and mouth ulcers so that we cannot exclude the U-shaped association between them. Contrary to treating mouth ulcers with estrogen, it had been identified that increased salivary estrogen levels leading to increased exfoliation of oral epithelium caused ulcerations during the normal menstrual cycle or pregnancy.^[5]^ Second, we may not completely exclude some unknown confounding factors in this article. Moreover, different people with different constitutions would bring about different relevant hormone levels, in this study we did not analyze the role of estradiol on mouth ulcers in terms of constitutions and age. Levels of estradiol vary greatly by age. Estradiol is secreted in small quantities during childhood in both sexes, and the secretion increases at puberty and reduces when old.^[7]^ So further studies should consider the effect of age in the relationship between estrogen and mouth ulcers. Third, after the screening, the number of SNPs for analysis was small, especially the representative female SNPs, and the population in this study was restricted to Europe. It remained to be investigated if our finding was consistent in other populations. Fourth, the SNPs representing exposures and outcomes were both derived from the UK biobank, there may be some overlapping samples that may bias MR estimates in the direction of observed associations. However, there is no clear way to evaluate the bias due to overlap.^[41]^ Although some used block jackknife resampling MR to mitigate bias, it can not directly identify the existence of horizontal pleiotropic SNPs that may lead to bias in causal inference. Meanwhile, the block jackknife resampling framework requires access to individual-level phenotype and genotype data that we did not have a good way to evaluate.^[42]^ Population overlapping could increase the possibility of false positives, but it was unlikely to affect our conclusion for our negative results.

In conclusion, this MR analysis provided no evidence of a causal association between decreased estradiol and the risk of mouth ulcers. The pathogenesis and treatments of mouth ulcers still need further studies like clinical trials and basic experiments to elucidate.

## Acknowledgments

We thank to IEU and UK Biobank consortiums for their sharing of GWAS summary data for our Mendelian randomization analyses.

## Author contributions

**Conceptualization:** Yaqian Zhang.

**Data curation:** Ruihanqiu Liu.

**Formal analysis:** Weiyu Liang.

**Project administration:** Weiling Qu, Yan Lu.

**Software:** Kunting Zhong.

**Visualization:** Kunting Zhong.

**Writing – original draft:** Yaqian Zhang.

**Writing – review & editing:** Weiyu Liang.

## Supplementary Material



**Figure:**