# Supplementary material for: Causal associations between estradiol and mouth ulcers: A Mendelian randomization study
Source: Medicine (Baltimore). 2024 Apr 26;103(17):e37989. doi: 10.1097/MD.0000000000037989 (PMC11049722; doi:10.1097/MD.0000000000037989)
Supplement: Supplementary file 1 [file medi-103-e37989-s001.docx]

| **Traits** | **Population** | **Sex** | **Sample Size**  **(case/controls)** | **Database** | **PubMed ID** |
| --- | --- | --- | --- | --- | --- |
| Depression | U.K. | NA | 27,568/457,030 | ebi-a-GCST90038650 | 33959723 |
| Anxiety or panic attacks | U.K. | NA | 6,514/478,084 | ebi-a-GCST90038651 | 33959723 |
| Reaction to severe stress and adjustment disorders | European | Males and Females | 182/337,017 | ukb-a-526 | -* |

*****The data source of Reaction to severe stress and adjustment disorders came from <https://gwas.mrcieu.ac.uk/datasets/ukb-a-526/>, NA, not available;

**Supplement table1** The information on confounders’ SNPs
